# Acute Silica Exposure Triggers Pulmonary Inflammation Through Macrophage Pyroptosis: An Experimental Simulation

**DOI:** 10.3389/fimmu.2022.874459

**Published:** 2022-04-07

**Authors:** Haoyu Yin, Lei Fang, Lifeng Wang, Yu Xia, Jiaqi Tian, Lan Ma, Jing Zhang, Ning Li, Weixiu Li, Sanqiao Yao, Lin Zhang

**Affiliations:** ^1^ Clinical Medical Research Center for Women and Children Diseases, Maternal and Child Health Care Hospital of Shandong Province, Shandong University, Jinan, China; ^2^ School of Public Health, Weifang Medical University, Weifang, China; ^3^ Department of Central Laboratory, Shandong Provincial Hospital Affiliated to Shandong University, Jinan, China; ^4^ School of Public Health, North China University of Science of Technology, Tangshan, China; ^5^ School of Public Health, Xinxiang Medical University, Xinxiang, China

**Keywords:** silica, macrophage, pyroptosis, ROS, NLRP3 inflammasome

## Abstract

Silica is an essential substrate of various materials, and inhaling silica induces pulmonary diseases potentially associated with macrophage pyroptosis. Utilizing silica of micro- and nano- sizes, we explored the role of macrophage pyroptosis in silica-induced pulmonary inflammation. Under the transmission electron microscopy, we found that the internalization of silica nanoparticle induced membrane rupture and increased the number of intracellular vacuoles, and both sizes of silica could suppress cell viability and proliferation. Also, silica-exposed macrophages generated higher levels of ROS, together with the upregulated expression of NLRP3, ASC, Caspase-1, GSDMD, IL-1β, and IL-6. However, the expression of these proteins was suppressed after removing ROS or NLRP3. In addition, we found increased expression of TLR4 and NF-κB responsible for silica recognition and pyroptosis priming after silica exposure. For *in vivo* studies, we established animal model by intratracheally instilling 5 mg of silica into mice with/without NLRP3 inhibition. Four weeks later, we found diffused infiltration of inflammatory cells and enhanced collagen hyperplasia partially reversed by additional treatment with MCC950, so as the expression of pyroptotic molecules and proinflammatory cytokines. In particular, the dual immunofluorescent staining showed co-expression of macrophage-specific biomarker F4/80 and NLRP3 within the cells, and silica of nano-size showed more potent toxicity and pathogenicity than that of the micro-sized particles both *in vitro* and *in vivo*. To sum up, macrophage pyroptosis is an upstream event of silica-induced pulmonary inflammation promoted by ROS through the TLR4/NLRP3/NF-κB signaling axis.

## Introduction

Silica has been immensely used in industry and daily life in the last few decades, and there are growing concerns about its related health and eco-environmental effects. Atmospheric silica particles are usually generated naturally, while industrial activities are becoming the leading route people exposing to silica particles (1). Generally, silica particles are internalized through respiratory and digestive tracts and skin, resulting in various physical disorders. Notably, the inhaled silica often induces pulmonary damage, shown as persistent pulmonary inflammation and even fibrosis in worse conditions, typical features of chronic obstructive pulmonary disease, asthma, and idiopathic pulmonary fibrosis (2, 3). In particular, long-term exposure to silica may cause silicosis, a severe occupational pulmonary disease threatening the lives of millions of workers worldwide (4).

Alveolar macrophage is a prominent participant in pulmonary immune response, and it plays an irreplaceable role in scavenging foreign bodies such as particles, fibers, and viruses. However, macrophage overstimulation causes immune response obstacles and destroys immune homeostasis, leading to over-release of proinflammatory cytokines and pulmonary inflammation and fibrosis (5). Pyroptosis is a newly defined proinflammatory and autolytic programmed cell death, and macrophage pyroptosis has been demonstrated to play essential roles in the pathogenesis of pulmonary inflammation (6). Usually, macrophage expresses pattern recognition receptors (PRRs) on their surface that bind the inhaled xenobiotics in a specific manner (7). For example, Toll-like receptor 4 (TLR4) is a representative PRRs responsible for recognizing silica particles, which pass silica invading signals to the nucleus and activate nuclear factor kappa-B (NF-κB) (8). Then, p65, a subunit of the activated NF-κB, upregulates NOD-like receptor pyrin domain-containing protein 3 (NLRP3) and precursors of proinflammatory cytokines such as Pro-interleukin-1β (Pro-IL-1β), interleukin-6 (IL-6), and interleukin-18 (IL-18), called pyroptosis priming (9, 10). Constant stimulation of silica particles often leads to NLRP3 inflammasome assembly consisting of NLRP3, apoptosis-associated speck-like protein containing CARD (ASC), and Pro-cysteinyl aspartate specific proteinase-1 (Pro-caspase-1). Just like a proteolytic enzyme, NLRP3 inflammasome hydrolyzes Pro-caspase-1 to form Caspase-1 and slices Gasdermin D (GSDMD) (11). Several P30 subunits generated from the N-terminal of GSDMD polymerize on the cell membrane and generate a non-selective ion channel, from which the majority of proinflammatory cytokines are released. Besides, silica exposure induces over synthesis of reactive oxygen species (ROS), while excessive ROS is potentially associated with oxidative damage-induced pyroptosis (12). Potentially, there may also be a crucial role for ROS in silica-induced macrophage pyroptosis.

Although sufficient evidence indicates silica-induced macrophage pyroptosis, a knowledge gap regarding the underlying molecular mechanisms still exists, shown as follows: is macrophage pyroptosis an indispensable segment in pulmonary inflammation? How does pyroptotic signal affect downstream events? And what is the role of ROS in silica-induced pyroptosis? Regarding these concerns, we established *in vitro* and *in vivo* pyroptosis models using silica of different sizes, through which we examined the effect of silica exposure on cell viability and biological function, and explored the molecular mechanism of silica-induced pyroptosis. Notably, we studied the role of ROS as a crucial segment between silica signal transduction and pyroptosis activation. Data obtained in this study would contribute to understanding the health effects of silica better, and contribute to the development of atmospheric silica particle exposure limits and the therapeutic strategy of related diseases.

## Materials and Methods

### Reagents

Glyceraldehyde-3-phosphate dehydrogenase (GAPDH) (GB6067) was used as a loading control, and Horseradish peroxidase-labeled goat-anti-rabbit immunoglobulin G (GB23303) was the secondary antibody, both of them along with high glucose Dulbecco’s modified Eagle cell culture medium (DMEM) (G4510-500ML) were purchased from Servicebio (Wuhan, China). Fetal bovine serum (FBS) (04-111-1A) was obtained from Biological Industries (Israel). Silica nanoparticles (NM000840), adenosine triphosphate (ATP) (C0550), and mitochondrial membrane potential assay kit (C2006) were purchased from Solarbio (Beijing, China). Silica dust of micro grade (P0176), TAK-242 (243984-11-4), and MCC950 (256373-96-3) were provided by Sigma-Aldrich (MO, USA). Primary antibodies, including NLRP3 (A5652), Caspase-1 (A0964), ASC (A16672), and IL-1β (A19635), were supplied by Abclonal (Wuhan, China). IL-6 antibody (DF6087) was provided by Affinity Biosciences (Cincinnati, OH, USA). Lipopolysaccharide (LPS) (S1732-25mg), cell counting kit 8 (CCK-8) (C0038), lactate dehydrogenase (LDH) assay kit (C0017), Calcein/Propidium iodide (PI) kit (C2015M), EdU with Alexa Fluor 555 kit (C0075S), Annexin V-FITC/PI detection kit (C1075S), ROS assay kit (S0033S), BAY 11-7082 (S1523-10mg), and N-acetyl-L-cysteine (NAC) (ST1546-10g) were purchased from Beyotime (Beijing, China).

### Cell Culture and Intervention

The ASC expressing murine macrophage cells RAW-ASC were purchased from InvivoGen (CA, USA) and were maintained in DMEM containing 10% FBS and 1% penicillin-streptomycin in a humidified atmosphere at 37°C with 5% CO_2_. The cells were passaged every 2~3 days and were harvested at the logarithmic phase. According to the requirements of different interventions, 2 × 10^4^ cells/well were seeded onto a 96-well plate and were divided into five groups: cells in the control group were maintained with DMEM; LPS group was treated with LPS (1 μg/mL) for 6 h; silica and positive control groups were pre-treated with LPS, then exposed to nano-silica at 100 μg/mL, micro-silica at 750 μg/mL, or ATP at 3 mM for 4 h, where the median lethal dose of silica was determined by CCK8 assay. For the mechanism study of silica-induced pyroptosis, different inhibitors were added to cells additionally: MCC950 (NLRP3 inhibitor, 10 μM), TAK-242 (TLR4 selective inhibitor, 1 mM), and BAY 11-7082 (NF-κB inhibitor, 20 μM). They were added ahead of LPS treatment 1 h; N-acetylcysteine (NAC) (ROS scavenger, 10 mM) was added before silica intervention 30 min.

### Transmission Electron Microscopy

After intervention with LPS for 6 h, RAW-ASC cells were exposed to nano- or micro-silica for 4 h. Then, the cells were fixed with 2.5% glutaraldehyde at 4°C for preservation and transportation. The fixed cells were centrifuged at 500 g for 5 min, and 0.1 M PB (pH 7.4) was added into the tube after the supernatant was discarded. Then, the precipitation was resuspended and washed in PB for 3min. After repeating the washing procedure three times, 1% cooled agarose solution was added to the EP tube. Before agarose solidification, the precipitation was suspended with forceps and wrapped in the agarose. Then, the cells were post-fixed in 1% OsO_4_ and dehydrated twice in a series of concentrations of ethanol (30%, 50%, 70%, 80%, 85%, 90%, 95%, 100%). After infiltration with EMBed for 12 h, the cells were embedded in epoxy resin, and the resin blocks were cut into slices of 60-80nm thin on the ultramicrotome. Then, the cell slices were fished onto the 150 meshes cuprum grids with formvar film and stained with 2% uranium acetate saturated alcohol solution for 8 min. Finally, the cells were subjected to transmission electron microscope (TEM) and the images were taken.

### CCK8 Assay

A total of 2 × 10^4^ RAW-ASC cells/well were inoculated onto a 96-well plate and intervened with the above procedures. Then, 10 μL of CCK8 reagent was added to each well and incubated at 37°C for 2 h. Finally, a modular multimode microplate reader (BioTek, USA) was utilized to measure the absorbance of the solution at 450 nm, and the alteration of cell viability was compared.

### EdU Assay

The proliferation of RAW-ASC cells was evaluated by EdU assay. First, 2 × 10^4^ cells/well were plated onto a 96-well plate and treated with different measures. Then, 20 μL of preheated 2 × EdU working solution was added, followed by incubation for 2 h. The cells were rinsed with phosphate-buffered saline (PBS) thrice, 5 min per time, and fixed with 100 μL of 4% paraformaldehyde 15 min at room temperature. After rinsing with the washing solution, 50 μL of 0.3% Triton X-100 was added to each well and incubated at room temperature for 10 min. Again, the cells were washed with PBS three rounds, and 50 μL of click additive solution was added and incubated in darkness for 30 min, followed by rinsing three rounds with PBS. Finally, 100 μL of 1 × Hoechst 33342 solution was added to each well. Ten min later, the cells were imaged with the ImageXpress^®^ micro confocal system. Those cells in the proliferation stage were labeled with red fluorescence and were quantified and compared by normalizing to cells in blue.

### LDH Release Assay

According to the manufacturer’s instructions, the cells were transferred onto a 96-well plate (2× 10^4^ cells/well) and treated with silica or ATP for different periods. Then, the cell culture supernatant was collected and centrifuged at 400 g for 5 min, and the cell debris was discarded. A mixture composed of 120 μL of supernatant and 60 μL of LDH working solution was prepared and added to a new plate, then incubated in darkness for 30 min at room temperature. The optical density (OD) was measured at 490 nm using a modular multimode microplate reader (Synergy H1, BioTek, USA).

### Calcein AM and PI Assay

A total of 2 × 10^4^ RAW-ASC cells/well were seeded onto a 96-well plate and subjected to the indicated treatments. 100 μL of Calcein AM/PI working solution were added, followed by incubation in darkness at 37°C for 30 min. Then, the cells were imaged with the ImageXpress^®^ micro confocal system where the living and dead cells were stained in green and red by Calcein AM and PI, respectively. The viability of macrophages was calculated by normalizing the PI-positive cells to (Calcein-positive cells + PI-positive cells) × 100%.

### Western Blot

A total of 2 × 10^6^ RAW-ASC cells/well were plated onto a 6-well plate and treated with specified interventions. The total protein was extracted with RIPA lysis buffer containing proteinase inhibitor cocktail and quantified using BCA kit. Ten μg of protein were separated by sodium dodecyl sulfate-polyacrylamide gel electrophoresis (SDS-PAGE) and transferred onto polyvinylidene fluoride (PVDF) membranes. After blocking in 5% non-fat milk at room temperature 1 h, the membranes were washed with TBST 3 times, 10 min per time, and incubated with primary antibodies at 4°C overnight. Subsequently, the membranes were rinsed 3 times with TBST, 10 min for each rinse, and hybridized with secondary antibody 1 h. After washing 3 times with TBST, the membranes were reacted with the electrochemiluminescence working solution and scanned under 457 nm and 520 nm emissions through the Fluor Chem HD2Gel imaging system (ProteinSimple, San Jose, CA, USA). The expression of these proteins of interest was quantified by normalizing to gray values of GAPDH. Independent experiments were performed at least three times, and the representative pictures were presented.

### Flow Cytometry Analysis

The integrity of cells was measured through Annexin V-FITC/PI dual fluorescent staining kit and quantified using flow cytometry. In line with the protocols above, 2 × 10^6^ cells/well were seeded onto a 6-well plate and subjected to the indicated treatments. The cells were harvested and washed with PBS, from which 5 × 10^4^ cells were separated and resuspended in 195 μL of Annexin V-FITC binding solution. Subsequently, 5 μL of Annexin V-FITC and 10 μL of PI were added to the cells in order. After incubation at room temperature for 15 min in darkness, the stained cells were placed on ice and subjected to the Accuri C6 flow cytometer (BD Biosciences, CA, USA). Data acquisition and analysis were performed using the BD software.

### Immunofluorescence Staining

A total of 2 × 10^4^ RAW-ASC cells were inoculated onto a 96-well plate, followed by the above-specified treatments. Those cells attached to the bottom of a 96-well plate were washed with PBS 3 times and fixed with 4% paraformaldehyde. Then, the cells were penetrated with 0.3% Triton X-100 for 10 min and blocked with goat serum at room temperature for 30 min. After discarding the serum, the cells were incubated with specific primary antibodies at 4°C overnight, then the secondary antibody with fluorescence was added, followed by 1 h incubation in darkness at room temperature. Finally, 10 μL of Hoechst was added to stain the nuclei in blue for 10 min, and the cells were subjected to the ImageXpress^®^ micro confocal system. The expression of specific proteins was observed and quantified by the fluorescence intensity between groups.

### ROS Determination

A total of 2 × 10^4^ RAW-ASC cells/well were transferred onto a 96-well plate and treated with specified interventions, from which 1 × 10^4^ cells were separated and incubated with 100 μL of diluted DCFH-DA probes at 37°C for 20 min. After rinsing with serum-free DMEM thrice, the OD values of the cells were measured using a modular multimode microplate reader (excitation wavelength 488 nm, emission wavelength 525 nm). Accordingly, the ROS levels were quantified and compared.

### Mitochondrial Membrane Potential Measurement

A total of 2× 10^4^ RAW-ASC cells/well were inoculated onto a 96-well plate, followed by the above-specified treatments. After washing with PBS once, 100 μL of JC-1 working solution pre-mixed with the same volume of DMEM was added to the cells. After incubation at 37°C for 20 min, the cells were washed twice with JC-1 buffer solution and suspended with 200 μL of serum-free DMEM. Finally, the JC-1-conjugated cells were imaged by the ImageXpress^®^ micro confocal system.

### Animal Maintenance and Intervention

The use of animals has been permitted by the ethics committee of Maternal and Child Health Care Hospital of Shandong Province, Shandong University, and proof of approval (No.2020-1) is available upon request. In total, 52 C57BL/6 mice (male, age 6-8 weeks, weighing 20-25 g) were purchased from the Medical Laboratory Animal Center, Weifang Medical University (Weifang, China) and placed in an observation chamber and given food and water. The animal maintenance conditions were strictly controlled at 20-24°C, with a standard 12 h light-dark cycle and a 54-59% relative humidity. The mice were allowed to adapt to conditions for at least seven days before the intervention. Accordingly, the mice were divided into four groups: mice in the control group were intratracheally instilled with 50 μL of saline, the nano-silica group had 50 μL of 100 mg/mL silica nanoparticles, the other two groups were additionally intraperitoneally injected with 15 mg/kg of MCC950, twice a week. The dose of silica was determined according to previous studies that had successfully established the experimental silicosis model. Four weeks later, all mice were anesthetized and sacrificed.

### Pathological Examination by Hematoxylin and Eosin (H&E) Staining

The lung tissues of mice were dissected, and the pathological changes were examined by H&E staining according to previous protocols (13). First, the lung tissues were fixed in 4% formaldehyde for 24 h, then dehydrated and embedded in paraffin. H&E staining was conducted on slices of the lung tissues, and the pathological changes were imaged and scanned with Pannoramic MIDI software (3DHIESTECH, Budapest, Hungary).

### Immunohistochemistry and Immunofluorescence

The lung tissues were prepared at approximately 4 μm thickness, and the paraffin around the lung tissues was cleared in xylene. Then, the tissues were hydrated in a series of concentrations of ethanol (100%, 95%, and 70%), 5 min per concentration, followed by rinsing three rounds with PBS at room temperature. The antigen retrieval was processed by blocking the lung tissues in 5% bovine serum albumin (BSA) solution for 30 min, then incubated with TLR4 and NF-κB for immunohistochemistry at 4°C overnight. Subsequently, the lung tissues were reacted with the biotinylated secondary antibody 1 h at room temperature, followed by washing with PBS 3 times. The tissue slices were immersed in DAB stock 3 min and counterstained using hematoxylin 2 min. After washing with tap water, the nuclei were stained in blue. For immunofluorescence, the antigen retrieved tissues were hybridized with fluorophore-conjugated NLRP3, GSDMD, and IL-1β antibodies for 1 h. After washing with PBS 3 times, the tissues were imaged using the ImageXpress^®^ micro confocal system. The expression of the protein was quantified using ImageJ software (NIH, MD, USA).

### Masson Staining and Resorcin-Fuchsin Staining

The lung tissues were prepared per the above description. After the clearance of paraffin, the lung tissues were soaked in Masson solution A at room temperature overnight, then incubated at 65°C for 30 min and washed with tap water 30 s. The tissues were steeped in Masson solution B and C for 1 min, and differentiated by 1% hydrochloric acid and alcohol for 1 min. Again, the tissues were stained with Masson solution D, E, and F in turn. Then, the tissues were rinsed and differentiated with 1% glacial acetic acid and dehydrated using absolute ethanol, 1-Butanol, and dimethylbenzene. For Resorcin-Fuchsin staining, the tissues were dipped into resorcin-fuchsin staining solution A, placed at room temperature for 4 h, and put into 1% hydrochloric acid alcohol differentiation solution for rapid differentiation for 2s, then washed and microscopically examined until transparent purple elastic fibers were observed. Similarly, the tissues were stained using resorcin-fuchsin staining solution B and resorcinol basic magenta staining solution C, then dehydrated in absolute ethanol. After sealing, the pulmonary collagen was imaged with Pannoramic MIDI (3DHIESTECH, Budapest, Hungary).

### Statistics

Data obtained in this study were collected, analyzed, and visualized using R v3.6.3 for Windows (Vienna, Austria). The differences between groups were compared using a two-tailed *t*-test, or one-way analysis of variance (ANOVA) where comparison was adopted between ≥ 3 groups followed by an SNK *post-hoc* test. The quantified results were visualized using bar charts, and a star was labeled when the difference was statistically significant at 0.05 level.

## Results

### Characterization and Cytotoxicity Evaluation of Silica Particles

**Figure 1:**
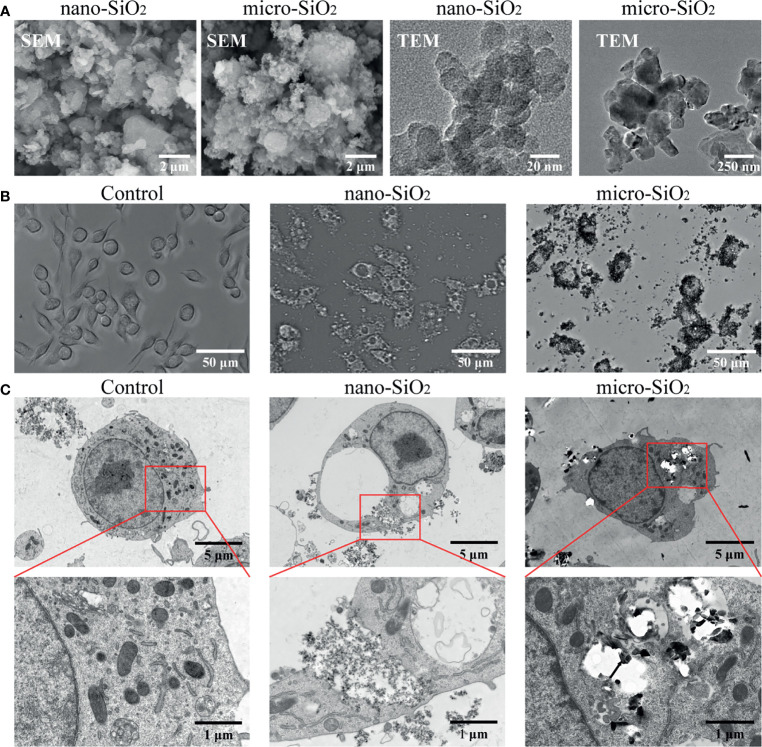
Characterization and internalization of silica particles *in vitro*. **(A)** Images taken by scanning electron microscopy (SEM) and transmission electron microscope (TEM) showing nano- and micro-silica particles. **(B)** Biodistribution of silica particles under optical microscopy. RAW-ASC cells are pre-treated with LPS for 6 h, then exposed to nano-silica at 100 μg/mL or micro-silica at 750 μg/mL for 4 h. **(C)** TEM images showing silica uptaken by RAW-ASC cells. Black arrow, silica particles; Red arrow, vacuoles; Yellow arrow, ruptured mitochondria.

The physicochemical properties of different sizes of silica particles were characterized through SEM and TEM. As shown in **Figure 1**, the TEM images showed that the aerodynamic diameters of nano- and micro-silica particles were approximate 30 nm and 500 nm. Both sizes of silica particles were of high dispersity under SEM. For particles applied to RAW-ASC cells, we found that different sizes of silica particles could be internalized intracellularly, causing cell membrane rupture, mitochondrial lysis, and the formation of vacuoles, especially in cells treated with nanoparticles (**Figure 1B**). In addition, compared with the control group, the number of nano-silica exposed cells declined significantly, and more membrane-invaginated cells scattered over the observation field were found, and the majority of them were covered in part by particles (**Figure 1**).

**Figure 2:**
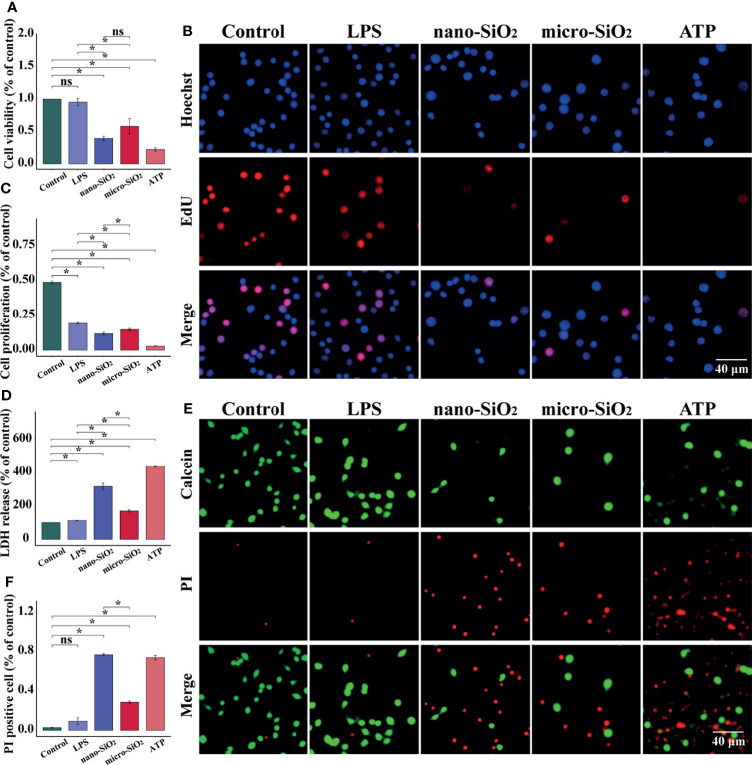
Silica suppresses the viability and proliferation of RAW-ASC cells. RAW-ASC cells are pre-treated with LPS for 6 h, then exposed to silica or ATP for 4 h. **(A)** CCK-8 assay showing the alteration of cell viability between groups. N=6. **(B)** Images of EdU assay showing the proliferation of RAW-ASC cells. Nuclei are stained in blue by DAPI, cells in the proliferation stage are stained in red by EdU. **(C)** Quantitative analysis and comparison of cell proliferation between groups. N=6. **(D)** LDH assay showing the release of LDH between groups. N=6. **(E)** Images of Calcein/PI staining showing the status of RAW-ASC cells. The living cells are stained in green by Calcein, and the ruptured cells are stained in red by PI. **(F)** Quantitative analysis and comparison of ruptured cells between groups. N=7. *P < 0.05; *ns, not significant*.

To evaluate the cytotoxicity of micro- and nano- silica particles, we examined the viability, proliferation, and membrane integrity of RAW-ASC cells. Compared with the control and LPS groups, we found a significant decrease in cell viability and proliferation after silica exposure, and a sharper decrease was determined in the ATP group, a positive control for pyroptosis (**Figure 2C**). In contrast, the release of LHD in silica-exposed macrophages was much higher than the control and LPS groups (**Figure 2**). Moreover, the ruptured macrophages were indicated *via* fluorescent staining method, by which PI could cross over the damaged cell membrane under pyroptotic conditions, while the healthy cells with integrated cell membrane were stained in green by Calcein. Comparing to the control and LPS groups, the PI-positive cells were highly detected after both sizes of silica exposure and ATP treatment (**Figure 2E**). In addition, the alterations caused by silica were always more robust than that of the mico-silica. These data suggest that exposure to silica suppresses the viability and proliferation of macrophages and promotes cell rupture and LDH release, resulting in pyroptosis-like alterations.

### Exposure to Silica Induces NLRP3-Dependent Macrophage Pyroptosis

**Figure 3:**
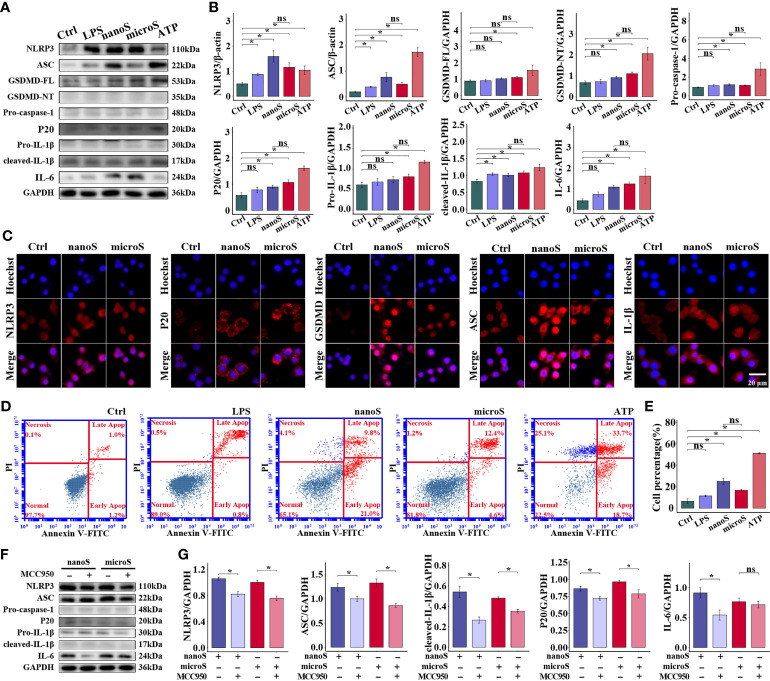
Silica exposure triggers pyroptosis of RAW-ASC cells. RAW-ASC cells are pre-treated with LPS for 6 h, then exposed to silica or ATP for 4 h. **(A)** Expression of NLRP3, ASC, GSDMD-FL, GSDMD-NT, Pro-caspase-1, P20, Pro-IL-1β, cleaved-IL-1β, and IL-6 in RAW-ASC cells. **(B)** Quantitative analysis and comparison of proteins in **(A)** The expression of these proteins is quantified by normalizing to β-actin or GAPDH. N=3. **(C)** Immunofluorescence images showing the expression of NLRP3, ASC, GSDMD, P20, and IL-1β in RAW-ASC cells. Red, target protein; Blue, nuclei. **(D)** Dot plot of flow cytometry showing the alteration of cell population after silica exposure. **(E)** Quantitative analysis and comparison of pyroptotic cells between groups. N=6. The Y-axis represents the total number of apoptotic cells. **(F)** Expression of NLRP3, ASC, Pro-caspase-1, P20, Pro-IL-1β, cleaved- IL-1β and IL-6. RAW-ASC cells are pre-treated with MCC950 for 1 h then LPS 6 h and silica 4 h. **(G)** Quantitative analysis and comparison of protein in **(F)** N=3. *P < 0.05; *ns, not significant*.

To reveal the underlying mechanism of silica-induced adverse effects on macrophages, we detected the expression of specific biomarkers related to pyroptosis. As shown in **Figure 3A**, compared with the control group, the expression of NLRP3, ASC, GSDMD, Caspase-1, IL-1β, and IL-6 was upregulated after ATP treatment, and the majority of these proteins showed an increasing trend after silica exposure, while micro-silica could not upregulate GSDMD of full length (FL). Notably, LPS induced upregulation of NLRP3, ASC, and cleaved IL-1β, and comparison between micro- and nano-silica groups indicated non-statistical alterations of these proteins. Moreover, we conducted the immunofluorescent staining assay to detect the expression of NLRP3, ASC, GSDMD, P20, and IL-1β. As shown by the enhanced fluorescence intensity in **Figure 3**, we found elevated expression of these proteins after silica exposure, especially cells exposed to silica nanoparticles. Given the GSDMD pore generated during pyroptosis, we checked the membrane integrity of macrophages as evidence for pyroptosis activation. As shown in **Figure 3D**, the PI-positive cells increased after silica and ATP treatments, but the difference was of non-statistical significance as compared to the LPS group.

Since multiple routes lead to pyroptosis, we examined the role of NLRP3 in silica-induced pyroptosis. After suppressing NLRP3 using MCC950, the expression of NLRP3, along with ASC, P20, and IL-1β, was downregulated as compared to cells merely treated with silica, but the expression of Pro-caspase-1 was not affected. Besides, the specificity of NLRP3-induced pyroptosis was checked by detecting the expression of NLRP1 and AIM2 at the protein level, consistently, we found that, compared with the control group, the expression of both proteins was not statistically changed (**Figure S5**). These results demonstrate that exposure to silica induces assembly of NLRP3 inflammasome and causes macrophage pyroptosis.

### NLRP3-Dependent Macrophage Pyroptosis Demands TLR4 Recognition and NF-κB Mediation

**Figure 4:**
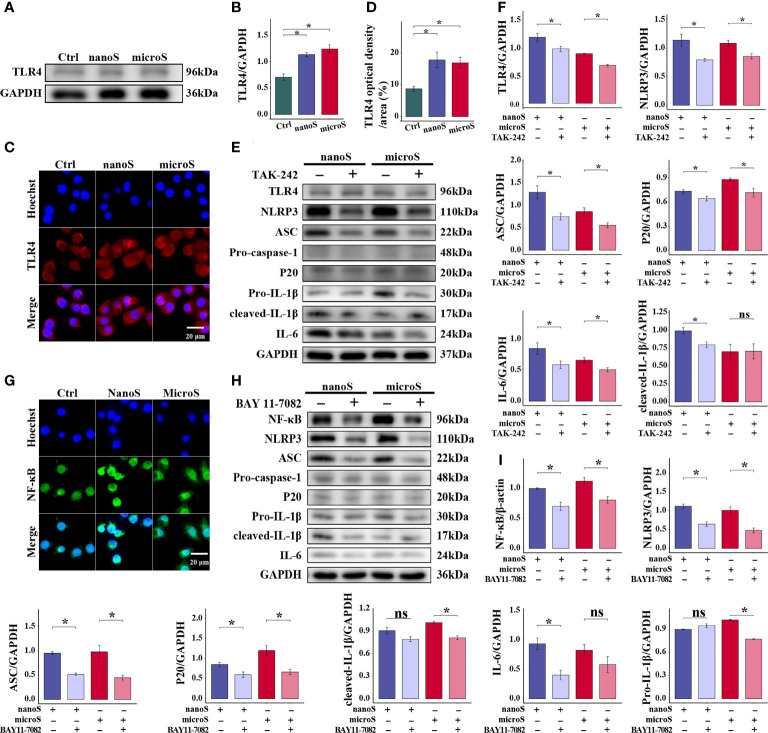
TLR4 and NF-κB are essential for silica-induced pyroptosis of RAW-ASC cells. RAW-ASC cells are pre-treated with LPS for 6 h, then exposed to silica for 4 h. **(A)** Expression of TLR4 in RAW-ASC cells determined by Western Blot. **(B)** Quantitative analysis and comparison of TLR4 by normalizing to GAPDH between groups. N=3. **(C)** Images of immunofluorescent staining showing the expression of TLR4 in RAW-ASC cells. TLR4 is stained in red; the nuclei are stained in blue. **(D)** Quantitative analysis and comparison of TLR4 between groups. N=5. **(E)** Expression of TLR4, NLRP3, ASC, Pro-caspase-1, P20, Pro-IL-1β, cleaved- IL-1β, and IL-6. RAW-ASC cells are pre-treated with TAK-242 for 1 h, then exposed to LPS for 6 h and silica for 4 h. **(F)** Quantitative analysis and comparison of target proteins in E by normalizing to GAPDH. N=3. **(G)** Images of immunofluorescent staining showing the expression of NF-κB in RAW-ASC cells. NF-κB is stained in green; the nuclei are stained in blue. **(H)** Expression of NF-κB, NLRP3, ASC, Pro-caspase-1, P20, Pro-IL-1β, cleaved- IL-1β, and IL-6. RAW-ASC cells are pre-treated with BAY 11-7082 for 1 h, then exposed to LPS for 6 h and silica for 4 h. **(I)** Quantitative analysis and comparison of protein in H by normalizing to β-actin or GAPDH. N=3. *P < 0.05; *ns, not significant*.

Precise recognition of xenobiotics is essential for innate immune response, where TLR4, an extensively studied pattern recognition receptor, has a wide response spectrum. As shown in **Figure 4A**, the expression of TLR4 was upregulated after silica exposure, along with an enhanced fluorescence intensity as compared to the control group, but there was no difference found between different sizes of silica particles. Using TAK-242 as a TLR4 inhibitor, we found that the expression of NLRP3, ASC, P20, and IL-6 were downregulated, while no statistical alteration was determined for Pro-caspase-1 and Pro-IL-1β between groups (**Figure 4**). In short, TLR4 is necessary for silica recognition and macrophage pyroptosis.

Furthermore, to explore the association of TLR4 with pyroptosis, we detected the expression of NF-κB, a classic nuclear factor functioned as a downstream responder of TLR4. As shown in **Figure 4G**, we found enhanced fluorescence intensity of NF-κB after silica exposure, especially in cells treated with silica nanoparticles. After treatment with BAY 11-7082, the expression of NF-κB was downregulated in both micro- and nano-silica groups, so as the expression of NLRP3, ASC, and P20. At the same time, the expression of Pro-caspase-1 and IL-1β was downregulated in the BAY 11-7082 + micro-silica group, and IL-6 was downregulated in the BAY 11-7082 + nano-silica group as compared to their corresponding controls. These data suggest that the activation of NF-κB is a follow-up event of silica recognition and showed in the upstream of NLRP3-mediated macrophage pyroptosis.

### ROS Accelerates TLR4/NF-κB/NLRP3-Mediated Macrophage Pyroptosis

**Figure 5:**
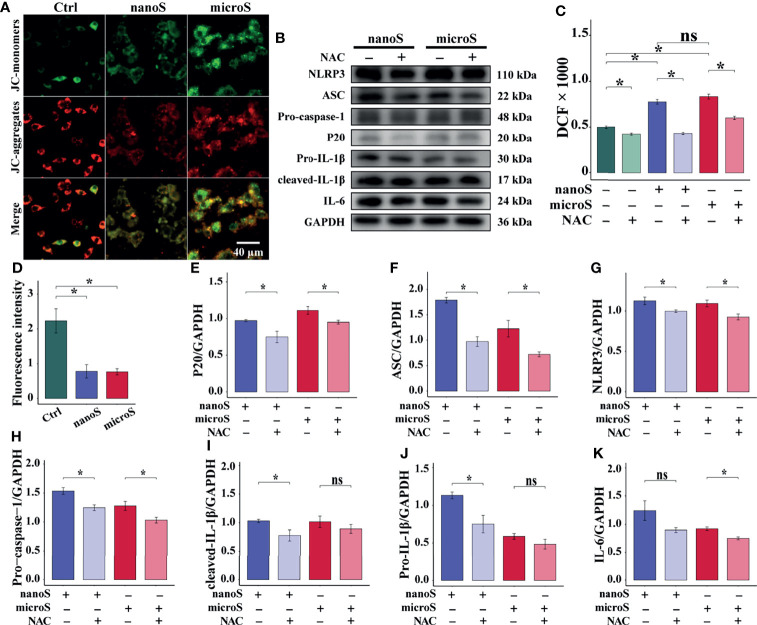
ROS promotes silica-induced pyroptosis in RAW-ASC cells. RAW-ASC cells are pre-treated with LPS for 6 h, then exposed to silica for 4 h. For NAC intervention, the cells are treated with NAC at 10 mM for 30 min in advance. **(A)** Immunofluorescence images showing JC-1-stained RAW-ASC cells, where the JC-1 aggregates (high potential) are stained in red; the monomers (low potential) are stained in green. **(B)** Expression of NLRP3, ASC, Pro-caspase-1, P20, Pro-IL-1β, cleaved-IL-1β, and IL-6. **(C)** Measurement and comparison ROS content between groups. N=8. **(D)** Quantitative analysis and comparison of fluorescence intensity of images in A between groups. N=4. **(E-K)** Quantitative analysis and comparison of protein in B by normalizing to GAPDH. N=3. *P < 0.05; *ns, not significant*.

ROS is believed to play crucial roles in a wide range of physical activities. Our previous work has also demonstrated the reproductive and developmental toxicity of oxidative stress in silica-exposed mice (14). To investigate the role of ROS in NLRP3-mediated pyroptosis, we removed intracellular ROS by NAC and observed on the alteration of mitochondrial membrane potential and the expression of pyroptotic molecules. As shown in **Figure 5**, compared with the control group, we found an elevated expression of ROS after silica exposure, and NAC could remove the over-generated ROS. Also, we found enhanced fluorescence intensity of JC-1 monomer (green) and diminished intensity of JC-1 aggregates (red), indicating the depolarization of the mitochondrial membrane potential after silica exposure.

To evaluate the effect of ROS on pyroptosis, we detected the expression of pyroptotic molecules and found suppressed expression of NLRP3, ASC, Pro-caspase-1, and P20 after NAC treatment. Besides, the downregulation of Pro-IL-1β and cleaved IL-1β was determined in NAC + nano-silica group, and IL-6 was in NAC + micro-silica group. These data suggest that exposure to silica induces over-generation of ROS, causing depolarization of mitochondrial membrane potential and leading to NLRP3-mediated macrophage pyroptosis.

### Silica-Induced Macrophage Pyroptosis Is Associated With Pulmonary Inflammation and Fibrosis *In Vivo*

**Figure 6:**
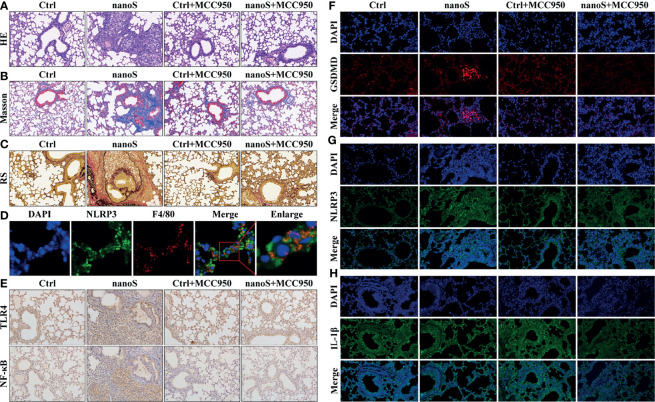
Silica induces macrophage pyroptosis and triggers pulmonary inflammation and fibrosis. **(A)** Images of hematoxylin-eosin (HE) staining showing the pathological changes of the lung tissues (20× magnification). The nuclei are stained in blue by hematoxylin, and the cytoplasm is stained in red by eosin. **(B)** Images of Masson staining showing the expression of collagen fibers (20× magnification). The collagen fibers are stained in blue, and the cytoplasm is stained in red. **(C)** Images of Resorcin-Fuchsin staining showing the expression of collagen fibers (20× magnification). The collagen fibers are stained in red; the muscle fibers are stained in purplish red, and other contents are stained in yellow. **(D)** Images of dual immunofluorescent staining showing the co-expression of NLRP3 and F4/80 within the cells. Blue, nuclei; Red, macrophage; Green, NLRP3. **(E)** Images of immunohistochemistry showing pulmonary expression of TLR4 and NF-κB (20× magnification). The positive areas are stained in brown. **(F–H)** Images of immunofluorescent staining showing the expression of GSDMD, NLRP3, and IL-1β (20 × magnification). The nuclei are stained in blue by DAPI, GSDMD is stained in red, NLRP3 and IL-1β are stained in green by specific antibodies.

To investigate the effect of macrophage pyroptosis *in vivo*, we established an experimental silica exposure model, observed lung tissue alteration, and detected the expression of pyroptotic molecules. As shown in **Figure 6**, compared with the control group, the infiltration of pulmonary inflammatory cells aggravated after silica exposure, the thickness of the alveolar wall increased, and the cell nodules composed of inflammatory cells diffused throughout the observation field. Masson and Resorcin-Fuchsin staining assay showed enhanced expression of collagen fibers as indicated by blue and red-stained areas, and collagen hyperplasia was widely detected in tissues with severe inflammation. After additional treatment of MCC950, the pulmonary inflammatory alteration and collagen hyperplasia were alleviated.

To locate the pyroptotic cells *in vivo*, we conducted the dual fluorescent staining assay using macrophage-specific antibody F4/80 and pyroptotic biomarker NLRP3. Interestingly, we found the co-stained cells within the cells, and these cells were more likely to be found in lung tissues of inflammation. Besides, we found upregulated expression of pyroptotic molecules such as NF-κB, TLR4, GSDMD, NLRP3, and IL-1β, and the upregulation could be effectively suppressed after MCC950 treatment. Data obtained *in vivo* jointly indicate that inhaling silica induces alveolar macrophage pyroptosis causally associated with pulmonary inflammation and fibrosis.

## Discussion

With the wide application of silica-based materials, the adverse effects of silica exposure have become a tough issue threatening both human beings and the eco-environment (15). To unveil the underlying mechanism of silica-induced physical disorders, we conducted this study by establishing *in vitro* and *in vivo* silica exposure models. For *in vitro* assay, we characterized the physicochemical properties of different sizes of silica particles and examined their toxicity on macrophages. In particular, we investigated the role of silica in triggering pyroptosis and its contribution to pulmonary inflammation. For *in vivo* studies, we observed the pathological changes of lung tissues after silica exposure and located the pyroptotic cells and detected the expression of pyroptotic molecules. Notably, we blocked the pyroptotic process using MCC950 and archived anticipated results. Data obtained in this study are of great significance on the evaluation of silica ecological toxicity and contribute to the prevention and treatment of related diseases.

Given the aerodynamic diameter of silica in the atmosphere, we exposed macrophages to both micro- and nano-silica particles, where the LD50 doses of silica were determined according to the CCK8 assay. Consistent with previous studies, we found both sizes of silica could suppress the viability and proliferation of macrophages. For silica of other sizes, Eun-Jung Parka reported that exposure to 20 nm or 50 nm silica for 24 h could also suppress cell viability and enhance ROS generation as compared to the control (16). Antonella Marrocco argued that the decrease of cell viability was associated with intracellular metabolic reprogramming, such as the enhanced uptake of glucose and aerobic glycolysis, suppressed complex I, and over-activated complex II (17). Herein, we showed more detailed information on silica-induced alterations, such as the appearance of intracellular vacuoles and morphology of the engulfed silica particles. According to studies in other fields, the vacuole was usually detected in aged cells, while the internalization of Libby amphibole asbestos led to cytoplasmic vacuoles (18). Therefore, the appearance of vacuole indicated the poor status of silica-exposed macrophages. Different from 30 nm silica particles, the 500 nm silica particles were engulfed by a small part, but a lower proliferation rate and a higher LDH release level were determined in these cells. Combined with the disrupted cell membrane, we might infer that micro-silica caused severe results than the 30 nm ones. In addition, Annexin V-FITC/PI staining was commonly used as an efficient method for apoptotic cell detection, and it was applied in compliance with Calcein/PI staining to show cell integrity here. We reported an increased number of PI-stained cells in both experiments that are of high similarity with the pyroptotic positive control (ATP group).

Extracellular recognition of silica particles is an initial step for intracellular signal transduction and cell response, where TLR4 was reported to dominate this biological process (19). After silica exposure, we found that the expression of TLR4 was upregulated, and similar phenomena were also detected in macrophages of silica-exposed rats. Besides, exposure to cadmium, bacteria, and copper oxide nanoparticles led to similar alterations (20–22). For the intracellular response to irritants, the p65 subunit of NF-κB was a commonly studied regulator responsible for the transcription of dozens of genes after translocating to the nucleus (23, 24). In resting cells, NF-κB was bound to an inhibitory protein-κB and retained in the cytoplasm; it could be activated when exposed to different cues such as LPS, leading to nuclear translocation of NF-κB p65 component, which contained the major transcriptional regulatory domain responsible for activating NF-κB corresponding genes (25, 26). In this study, we visualized the entry of the p65 subunit into the nucleus *via* immunofluorescent staining assay, exhibiting convincing evidence for the priming of pyroptosis. Like pathogen-associated molecular patterns (PAMPs), silica was a second signal initiating the assembly of the NLRP3 inflammasome, consisting of NLRP3, ASC, and Pro-caspase-1 (27).

Various sources were generating ROS intracellularly, among which mitochondrion was the most important one that had been demonstrated to play crucial roles in inflammasome assembly (28). As characterized by previous studies, there was a tight association of mitochondrial adaptor protein with NLRP3, where mitochondrial adaptor protein was responsible for depolarization of mitochondrial membrane potential under excessive ROS conditions, and the activated NLRP3 could be bound to ASC and Pro-caspase-1 to initiate the assembly of NLRP3 inflammasome (29). Consistently, we found elevated ROS content in silica-exposed macrophages, along with the upregulation of NLRP3, ASC, Pro-caspase-1, suggesting a causal relationship between silica exposure, ROS elevation, NLRP3 inflammasome assembly, and pyroptosis.

GSDMD was an emerging target newly identified in the downstream of pyroptosis, and the P30 fragment (GSDMD-NT) of GSDMD could embed into the cell membrane and generate a non-selective ion channel, causing an efflux of proinflammatory cytokines and loss of hypertonic advantage of cells, resulting in cell swelling and rupture (30). Therefore, the release of IL-1β, IL-6, and IL-18 *via* GSDMD pore was commonly regarded as the terminal events and hallmarks of pyroptosis (31). Functionally, IL-1β was a potent promoter of inflammation, especially in the vasodilation and immune cell extravasation, that could amplify the inflammatory signals substantially (32, 33). IL-6 was highly expressed in the tumor microenvironment; it could enhance the secretion of IL-1β and IL-18 and trigger the inflammatory storm *in vivo* (34). This study exhibited an elevated expression of GSDMD after silica exposure, particularly in cells with ruptured membranes. Combined with the elevated levels of proinflammatory cytokines, here we provided convincing evidence for the initiation of silica-induced macrophage pyroptosis.

**Figure 7:**
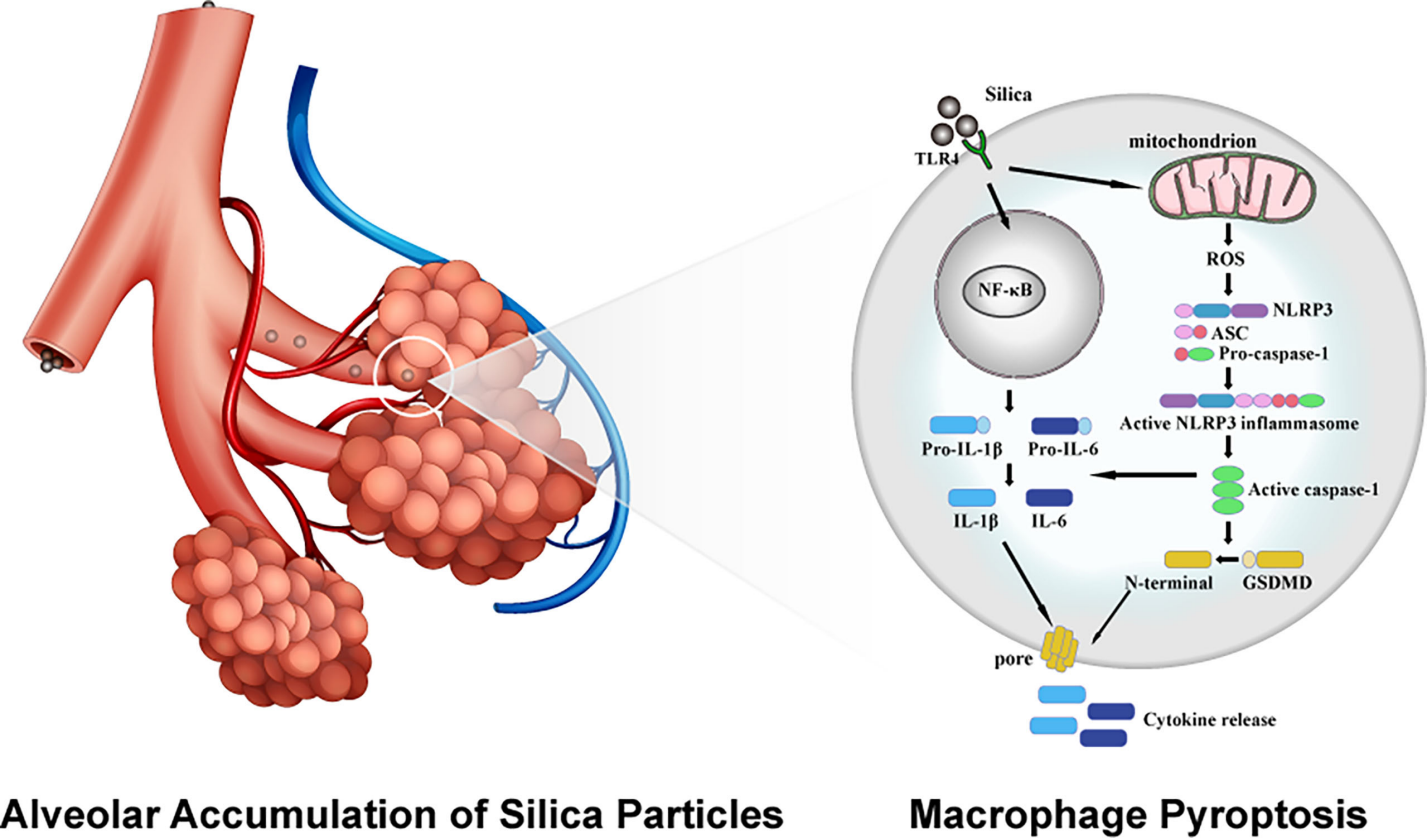
Schematic diagram of molecular mechanism of silica-induced macrophage pyroptosis.

To sum up, this study explored the biological toxicity of different sizes of silica particles comprehensively, where exposure to LD50 doses of micro- and nano- silica suppressed the viability and proliferation of macrophages, causing cell membrane rupture and release of proinflammatory cytokines. The in-depth study suggested a crucial role of macrophage pyroptosis in silica-induced pulmonary inflammation and fibrosis, as evidenced by the elevated ROS content and the enhanced expression of inflammatory cytokines. Utilizing specific inhibitors against ROS, NLRP3, TLR4, and NF-κB, the initiation of macrophage pyroptosis is encapsulated into three stages: TLR4-mediated recognition of silica, NF-κB-dominated intracellular signal transmission and pyroptotic priming, assembly of NLRP3 inflammasome and the activation of pyroptosis (**Figure 7**). These data highlight an emerging insight into the mechanistic study and clinical treatment of silica-induced pulmonary diseases, as well as contribute to the formulation of silica exposure limits and atmospheric silica management strategies.

## Data Availability Statement

The raw data supporting the conclusions of this article will be made available by the authors, without undue reservation.

## Ethics Statement

The animal study was reviewed and approved by Maternal and Child Health Care Hospital of Shandong Province, Shandong University (No.2020-1).

## Author Contributions

All authors contributed to the study conception and design. Project administration, Methodology, Data curation, Writing - original draft by HY. Investigation, Validation by LF. Validation by YX. Resources by LW. Methodology, Formal analysis, Project administration by JT. Investigation, Validation by LM. Investigation, Validation by JZ. Resources by NL. Resources by WL. Writing-review and editing by SY. Conceptualization, Resources, Supervision, Writing-review and editing, Funding acquisition by LZ and all authors commented on previous versions of the manuscript. All authors read and approved the final manuscript.

## Funding

This work was supported by the National Natural Science Foundation of China (82003405), the Natural Science Foundation of Shandong (ZR2020QH290), and the Project of Medical and Health Technology Development Program in Shandong Province (2018WS059). The funders had no role in study design, data collection, and interpretation, or the decision to submit the work for publication.

## Conflict of Interest

The authors declare that the research was conducted in the absence of any commercial or financial relationships that could be construed as a potential conflict of interest.

## Publisher’s Note

All claims expressed in this article are solely those of the authors and do not necessarily represent those of their affiliated organizations, or those of the publisher, the editors and the reviewers. Any product that may be evaluated in this article, or claim that may be made by its manufacturer, is not guaranteed or endorsed by the publisher.
