# Supplementary material for: Acute Silica Exposure Triggers Pulmonary Inflammation Through Macrophage Pyroptosis: An Experimental Simulation
Source: Front Immunol. 2022 Apr 7;13:874459. doi: 10.3389/fimmu.2022.874459 (PMC9021383; doi:10.3389/fimmu.2022.874459)
Supplement: Supplementary file 1 [file DataSheet_1.docx]

**Supplementary Files**


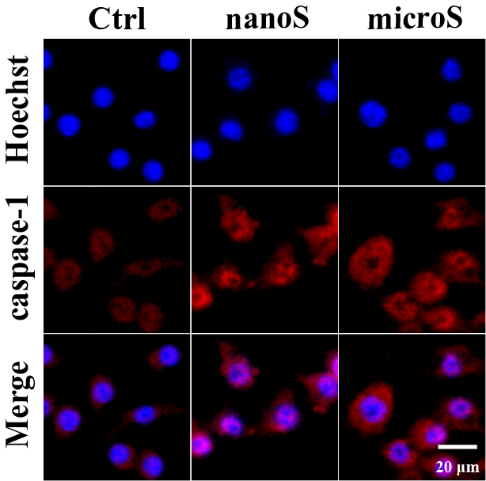


**Fig. S1.** Immunofluorescence images showing the expression of Caspase-1 between groups in Figure 3C. Red, Caspase-1; Blue, nuclei; nanoS, silica nanoparticle; microS, silica microparticle.


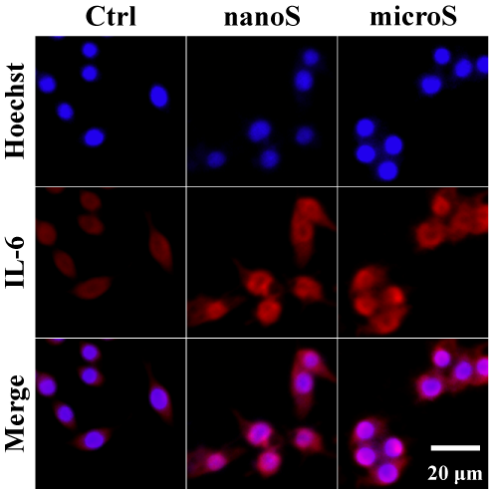


**Fig. S2.** Immunofluorescence images showing the expression of IL-6 between groups in Figure 3C. Red, IL-6; Blue, nuclei; nanoS, silica nanoparticle; microS, silica microparticle.


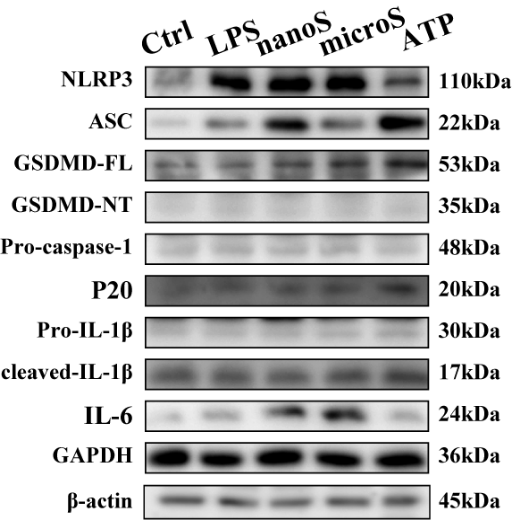


**Fig. S3** Figure 3A with β-actin.


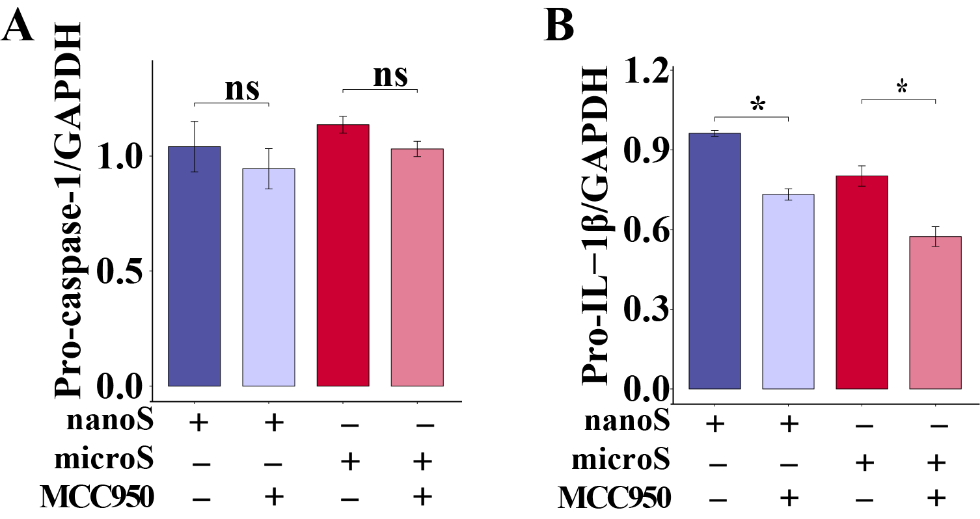


**Fig. S4** Quantitative analysis and comparison of Pro-caspase-1 (A) and Pro-IL-1β (B) in Fig. 3F. nanoS, silica nanoparticle; microS, silica microparticle; **p < 0.05*; *ns, not significant.*


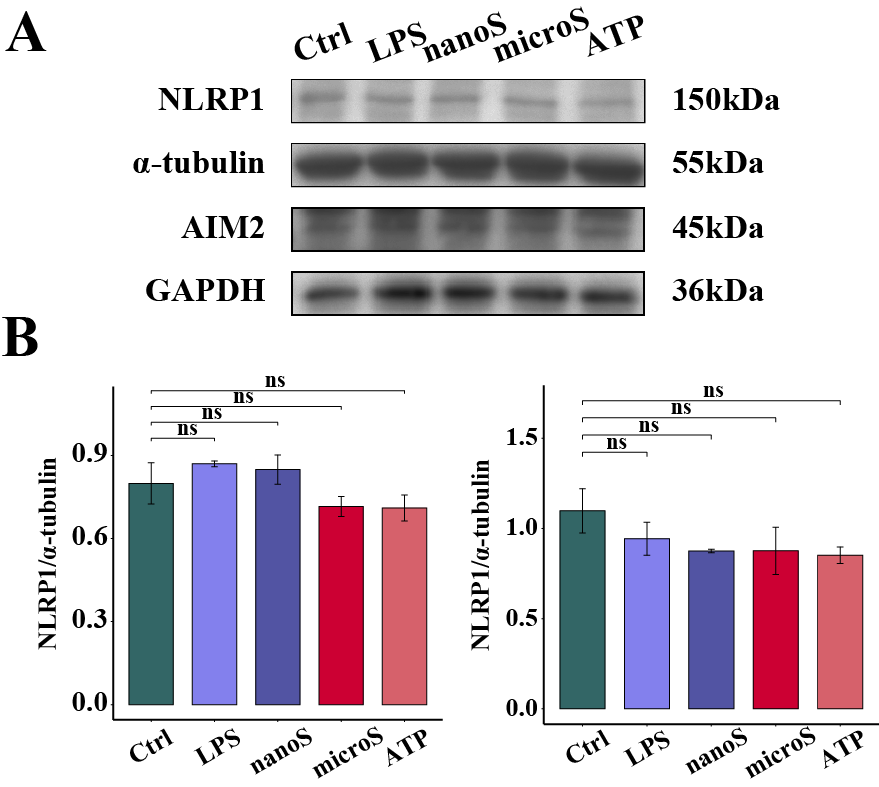


**Fig. S5** Expression of NLRP1 and AIM2 in RAW-ASC cells. RAW-ASC cells are pre-treated with LPS for 6 h, then exposed to silica or ATP for 4 h. (A) Expression of NLRP1 and AIM2. (B) Quantitative analysis and comparison of NLRP1 and AIM2. The expression of these proteins was quantified by normalizing to α-tubulin or GAPDH. N=3. *ns, not significant*.


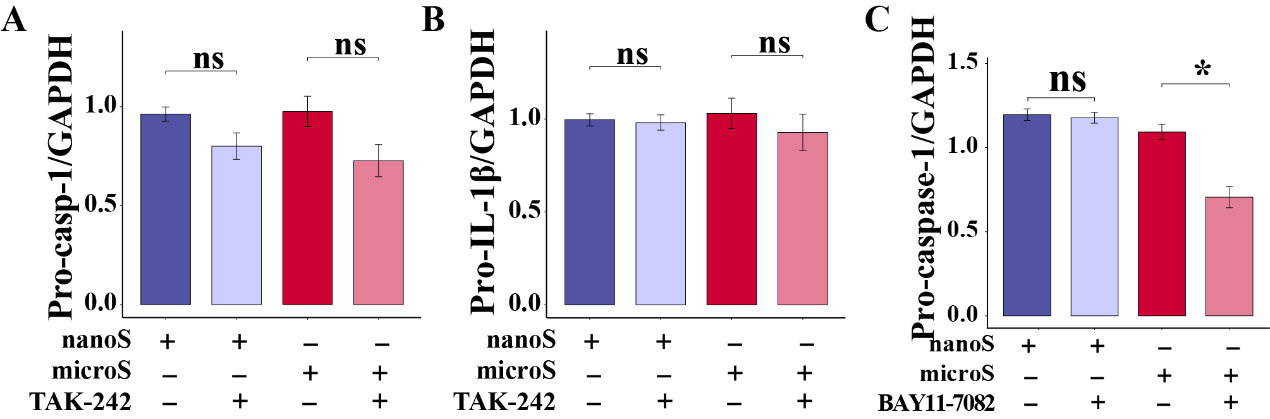


**Fig. S6** Quantitative analysis and comparison of Pro-caspase-1 (A, C) and Pro-IL-1β (B) in Fig. 4. nanoS, silica nanoparticle; microS, silica microparticle; **p < 0.05, ns, not significant.*


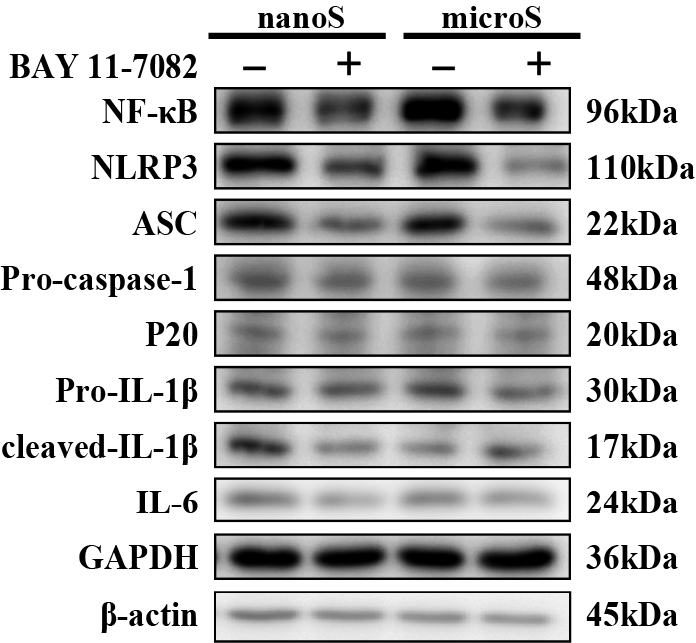


**Fig. S7** Figure 4H with β-actin.


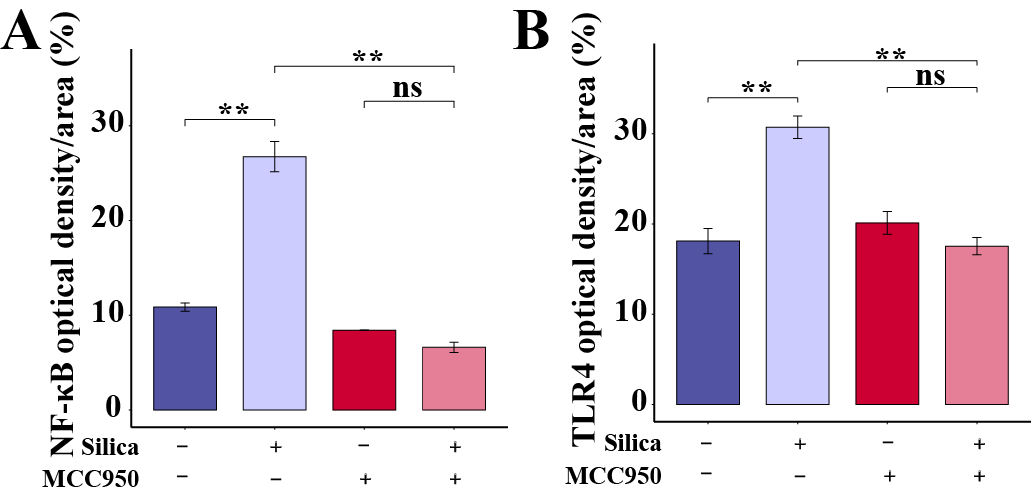


**Fig. S8** Quantitative analysis and comparison of NF-κB (A) and TLR4 (B) in Fig. 6E. nanoS, silica nanoparticle; microS, silica microparticle; **p < 0.05; ns, not significant.*
